# Examining healthcare professional delivery of health behaviour change interventions during a public health emergency: A multi-professional survey among NHS healthcare professionals

**DOI:** 10.1177/13591053241291478

**Published:** 2024-11-29

**Authors:** Chris Keyworth, Judith Johnson, Christopher J Armitage, Katharina Sophie Vogt, Tracy Epton, Mark Conner

**Affiliations:** 1 University of Leeds, UK; 2 University of Manchester, UK; 3 Manchester University NHS Foundation Trust, UK

**Keywords:** communication, health behaviour, health care, health care systems, health promotion

## Abstract

This study aimed to assess the extent to which healthcare professional characteristics and perceptions of major stressors during a public health emergency were associated with delivering health behaviour change interventions. A survey was administered in 2022 to a representative sample of 1008 healthcare professionals working in the UK’s National Health Service (NHS). Data were analysed using descriptive statistics and hierarchical linear regression. Older respondents, higher levels of job satisfaction, being a nurse or health visitor, and reporting higher levels of perceived impacts of the COVID-19 public health emergency were associated with higher prevalence of delivering interventions. Higher levels of emotional job stress were associated with greater time spent delivering interventions (but not with a higher prevalence of contacts involving intervention delivery). Interventions targeted at younger healthcare professionals, those reporting lower job satisfaction, and healthcare professionals other than nurses or health visitors would be particularly beneficial.

## Background

Unhealthy behaviours (e.g. engaging in risk behaviours such as smoking or failing to engage in protection behaviours such as physical activity) are important risk factors for long-term health conditions such as cardiovascular diseases, diabetes, and cancer, highlighting the global importance of promoting health behaviour change (World Health Organization, 2023). Public health policies, such as the Making Every Contact Count policy in the UK’s National Health Service (Public Health England, 2016), are used internationally to compel healthcare professionals to deliver health behaviour change interventions (e.g. reducing alcohol intake, improving diet, increasing physical activity, and smoking cessation) during routine consultations with patients (Keyworth et al., 2018; Meade et al., 2022; The Royal Australian College of General Practitioners, 2020; Whitlock et al., 2002). Behaviour change interventions can take many forms but the intervention encouraged by the MECC policy involves the delivery of health promotion advice, such as advice to reduce alcohol intake or increase physical activity. As a minimum, all healthcare professionals who have direct contact with patients are advised to ‘raise awareness, motivate and signpost people to help them improve their health and wellbeing’ (Public Health England, 2016). Due to their frequent patient contact, healthcare professionals are an expected and trusted source of behaviour change advice (Keyworth et al., 2020, 2021; McPhail and Schippers, 2012; Whitlock et al., 2002). Further, regardless of their specialism, and also when considered outside of their professional remit, healthcare professionals appear to value providing behaviour change interventions as an important clinical activity (Keyworth et al., 2019), and patients welcome health behaviour change interventions during routine consultations (Keyworth et al., 2021). However, there was a recent decline in the proportion of routine consultations involving the delivery of behaviour change interventions (Keyworth et al., 2024) .

Public health emergencies of international concern (PHEIC) (WHO, 2016) place healthcare systems under considerable strain. The outbreak of novel infectious diseases, for example, requires rapid changes to the way healthcare is delivered, and consequently presents a number of major stressors (Kobres et al., 2019; Wilder-Smith and Osman, 2020). The most recent public health emergency, the COVID-19 pandemic, had significant impacts on healthcare professionals and organisations. Internationally, this has included understaffing (Lasater et al., 2021), a perceived fear of becoming infected with the virus (Liu et al., 2020), and dealing with a lack of personal protective equipment (Tabah et al., 2020). In the UK, research suggests healthcare professionals have faced a number of challenges during the pandemic, including inadequate training, a lack of consistent guidelines with respect to caring for patients during the pandemic, and a changing and challenging work environment (Al-Ghunaim et al., 2021; Hoernke et al., 2021), including a rapid shift to remote consultations (Greenhalgh et al., 2020; Murphy et al., 2021). Consequently, this may have led to changes in the way behaviour change interventions are delivered, as they may have become less of a priority during routine consultations.

There may be a number of potentially important social and psychological impacts of public health emergencies on healthcare professionals, which may help to explain the reasons for the decline in their delivery of behaviour change interventions (Keyworth et al., 2024) . It is widely recognised that healthcare professionals are known to report high levels of stress, depression and anxiety (Arora et al., 2022), and this is consistent with findings in the context of public health emergencies, where healthcare professionals have greater levels of psychological distress during periods where healthcare systems are placed under significant strain (Kisely et al., 2020). More recent research suggests stressors related to public health emergencies may lead to increased emotional exhaustion and stress (Ching et al., 2021; Couarraze et al., 2021; Moreno-Jiménez et al., 2021; Olaya et al., 2021), and higher levels of burnout in these groups (Huo et al., 2021).

In the context of the most recent public health emergency, COVID-19, specific measures have been used to further examine the effects of the pandemic on healthcare professionals. For example, studies report elevated levels of fear of COVID-19 amongst healthcare professionals (Çağış and Yıldırım, 2023; Zangeneh Soroush et al., 2022), and the perceived impacts of COVID-19, particularly amongst patient-facing healthcare professionals (Debski et al., 2021). Findings from a longitudinal survey of frontline healthcare professionals (specifically nursing and midwifery staff) showed high levels of negative psychological effects because of COVID-19, including concerning prevalence of post-traumatic stress disorder, stress, and anxiety (Couper et al., 2022). Studies have also reported that perceived fear of COVID-19 is also negatively associated with job satisfaction (Labrague and de Los Santos, 2021). However, to our knowledge, no research to date has examined the extent to which these factors may act as barriers to delivering health behaviour change interventions. It is crucial to understand the predictors of healthcare professional delivery of health behaviour change interventions to: (a) inform the development of interventions to support practice in the recovery from COVID-19 and (b) to prepare for how to maintain practice in the event of future public health emergencies.

Given the added pressures of public health emergencies, it would be valuable to examine which factors influence healthcare professionals’ delivery of behaviour change interventions. Whilst there is evidence from previous studies that social and psychological impacts of public health emergencies may impact on healthcare professionals themselves, to our knowledge, no research to-date has examined whether these drivers affect the delivery of behaviour change interventions in a post-COVID-19 NHS context. This is important because this may allow targeted interventions to be delivered to enhance the social and psychological drivers of healthcare professional behaviour. Consequently, this study aimed to assess the extent to which healthcare professional characteristics and perceptions of the social and psychological impacts of COVID-19 were associated with delivering health behaviour change interventions during routine consultations.

## Methods

### Design and procedure

A cross-sectional survey design was used. Healthcare professionals with a patient-facing role were recruited via a survey panel company (YouGov). A purposive sample of healthcare professionals intended to be representative of the National Health Service (NHS) workforce in the United Kingdom was invited to take part in an online questionnaire and were incentivised in accordance with YouGov’s points system, whereby respondents accumulate points for taking part in online surveys, which can be exchanged for cash or entry into a prize draw. The data were collated by YouGov and sent securely to the research team for analysis.

#### Transparency and openness

We describe our sampling plan, data inclusion criteria, and all measures in the study. Relevant data and code can be made available upon request. Data were analysed using SPSS Statistics 27. The study’s design and analyses were not preregistered. Ethics approval was obtained from a University Research Ethics Committee (ref: PSYC-398).

### Participants

The survey was conducted in the UK in February–March 2022 (a time which preceded a COVID-19 ‘peak’ when one in 23 people in the UK had the virus (up from 1 in 70 in December 2021) (Statistics OfN, 2023) in a sample of 1008 healthcare professionals working in the NHS. The survey was part of a wider questionnaire aimed at developing and piloting a theory-based intervention for healthcare professionals. A range of patient-facing healthcare professionals were recruited and included: general practitioners (GPs); specialist doctors; nurses; midwives, and scientific, therapeutic and technical staff (e.g. pharmacists, psychologists, speech and language therapists). The sampling frame aimed to obtain the widest possible variation in participants according to demographic characteristics.

### Measures

The questionnaire, as part of a wider survey (Keyworth et al., 2024) collected demographic information such as gender and age, and healthcare setting (e.g. primary care, secondary care).

#### Experiences delivering behaviour change interventions during routine practice

Participants were asked about the extent to which they delivered health behaviour change interventions as part of routine healthcare. Participants were asked to rate (using a 0%–100% rating scale): (a) what proportion of patients they saw who they perceived would benefit from health behaviour change interventions, (b) the proportion of times they delivered health behaviour change interventions to the patients they thought would benefit, and (c) how much of their contact time they spent delivering health behaviour change interventions to the patients they thought would benefit.

#### Capturing healthcare professional wellbeing

Overall job stress was measured using six items relating to employees’ experience of overall job stress. The six items scale (α = 0.94) comprised of: (a) three items adapted from Warr’s (1990) anxiety–contentment scale, based on the answers to the question: ‘over the last month, how much of the time has your job made you feel…?’, for each of three negative items – ‘tense’, ‘worried’, and ‘uneasy’, and three items adapted from Warr’s (1990) depression–enthusiasm scale, based on answers to the question ‘over the last month, how much of the time has your job made you feel…?’, for each of three negative items – ‘depressed’, ‘gloomy’ and ‘miserable’. Responses were coded on a seven-point Likert scale (*never* [1]–*all of the time* [7]). A total score was calculated, with a higher score indicating greater levels of overall job stress (range 6–42) (Deepchand et al., 2013; Lai et al., 2015).

Job satisfaction was measured using the Warr-Cook-Wall Job Satisfaction Scale (Warr et al., 1979), which includes 15 items (α = 0.93) measuring aspects of job satisfaction, comprising of intrinsic factors (e.g. freedom to choose own method of working) and extrinsic factors (e.g. physical work conditions). Responses were coded on a seven-point Likert scale (*extremely dissatisfied* [1]–*extremely satisfied* [7]). A total score was calculated, with a higher score indicating greater job satisfaction (range 15–105).

#### Capturing experiences of COVID-19

The psychological impact of COVID-19 was measured using two scales. First, the Impact of Events Scale with Modifications for COVID-19 (IES-COVID19) (Vanaken et al., 2020) was used to examine levels of trauma-related stress following COVID-19. The scale is based on the Impact of Events Scale (IES), a validated tool for the assessment of subjective distress following a traumatic event (Horowitz et al., 1979). Vanaken et al. modified the original IES to capture COVID-19, and the questionnaire has good test-retest reliability and internal consistency (Vanaken et al., 2020). The scale comprises 15 items (α = 0.93), each measured on a four-point Likert scale. Respondents are asked to rate the frequency of each event occurring over the previous week ([0] *not at all*, [1] *seldom*, [3] *sometimes*, [5] *often*). A total score was calculated, with a higher score indicating more severe perceived distress (range 0–75). As suggested for the IES scale, respondents can be categorised into four ranges: ‘subclinical’ (scores 0–8), ‘mild’ (scores 9–25), ‘moderate’ (scores 26–43) and ‘severe’ (scores ≥44); a score of 26 indicates clinically significant trauma symptoms.

Second, healthcare professionals completed The Fear of Coronavirus-19 Scale (Ahorsu et al., 2022), which assesses participants’ agreement with seven items (α = 0.90; e.g. ‘I cannot sleep because I am worried about getting coronavirus-19’) with respect to fear of COVID-19. Participants were asked to respond on a 5-point scale (strongly disagree [1]–strongly agree [10]). A total score was calculated (range 7–35), with higher scores corresponding to higher perceived fear of COVID-19. Previous research has suggested a two-factor model of the Fear of Coronavirus-19 Scale (Stone and Wang, 2023), with two distinct corresponding sub-scales (emotional fear reactions; e.g. ‘It makes me uncomfortable to think about the coronavirus’, and symptomatic [or physiological] expressions of fear; e.g. ‘My hands become clammy when I think about the coronavirus’). Bitan et al. (2020) found that a two-factor model explains a large proportion of the total variance observed in reported COVID-19-related fear (53.71 and 12.05% respectively). Therefore, scores were also calculated for the two corresponding subscales: emotional fear reactions and symptomatic expressions of fear (respective Cronbach’s alpha scores were good; 0.863 and 0.894, respectively).

### Analyses

Descriptive statistics were used to summarise sociodemographic variables, MECC-related activities (perceptions of the proportion of patients that would benefit from behaviour change interventions, the proportion to whom healthcare professionals deliver interventions, the amount of time spent on this activity), and the social and psychological impacts of the COVID-19 pandemic (impact of events, overall job stress, job satisfaction and fear of COVID-19). A series of hierarchical linear regressions were used to examine associations between sociodemographic factors, psychosocial variables, and delivery of behaviour change interventions. Perceived Impact of Events and both sub-scales of perceived fear of COVID-19 were entered into model 1 in this analysis, and age, gender, healthcare professional group (nurses and health visitors vs ‘other’), job stress, and job satisfaction were added in model 2. Model 3 tested for any significant interactions between the predictors in model 1 and the variables added in model 2 (with simple slopes analyses (Aiken and West, 1991) used to explore any significant interactions at low (mean –1 SD), mean, and high (mean +1 SD) levels of the moderator). For the purposes of the hierarchical linear regression model, healthcare professionals were divided into two groups. Nurses and health visitors were included in the first group, and all other healthcare professionals were grouped into the second group. This approach was taken because (1) linear regression only allows for comparisons between two groups when variables are nominal and (2) nurses and health visitors were the single largest group in our sample. Although the three main outcomes were strongly intercorrelated (rs > 0.55) each was of interest in their own right and therefore three separate hierarchical linear regression models were conducted for each of the three dependent variables: perceptions of patient benefit of interventions, delivery of interventions, and time spent delivering interventions. Each model was adjusted for potential correlates of delivering interventions (age, gender and ethnicity). These regression analyses were repeated for the three main outcomes.

## Results

### Participant characteristics

The total sample (*n* = 1008) included nurses and health visitors (*n* =  409), specialist doctors (*n* =  146), scientific, therapeutic, and technical staff (*n* = 143), other HCHS staff (*n* = 121), healthcare professionals providing support to clinical staff (*n* = 58), nurses working in GP practices (*n* = 46), general practitioners (*n* =  45), midwives (*n* = 30), and ambulance staff (*n* = 10). Healthcare professionals mostly worked in primary care (*n* = 193), acute care (*n* = 414) or community care (*n* = 230). Most healthcare professionals were White British (87.8%), almost three-quarters were women (73.5%), with a mean age of 45 years (SD = 11.97). An overview of our sample is presented in Table 1.

**Table 1.** Sample characteristics.

Variable	*n*	(%)	Mean	(SD)
Gender				
Men	266	(26.4)		
Women	741	(73.5)		
Age, years			45	(11.97)
Ethnicity				
White	885	(87.8)		
Black, Asian, minority ethnic/prefer not to say	116	(11.5)		
Healthcare professional group				
General practitioners	45	(4.5)		
Specialist doctors	146	(14.5)		
Nurses and health visitors	409	(40.6)		
Midwives	30	(3.0)		
Ambulance staff	10	(1.0)		
Scientific, therapeutic and technical staff	143	(14.2)		
Nurses working in GP practices	46	(4.6)		
Support to clinical staff	58	(5.8)		
Other HCHS staff/unknown classifications	121	(12.0)		
Total				
Setting				
NHS acute care	414	(41.1)		
NHS tertiary care	89	(8.8)		
NHS community care	230	(22.8)		
NHS primary care	193	(19.1)		
Other	82	(8.2)		
Of the service users you see in a typical working week, *what proportion do you think would benefit* from you making every contact count?			49.11	(35.64)
Of the service users you see in a typical working week, who you think would benefit, *with what proportion* do you make every contact count?			38.00	(36.33)
Of the service users you see in a typical working week who you think would benefit, *how much of their appointment time do you spend* with them making every contact count?			26.54	(32.68)
Impact of events			20.03	16.83
Subclinical (scores 0–8)	319	(31.6)		
Mild (scores 9–25)	338	(33.5)		
Moderate (scores 26–43)	244	(24.2)		
Severe (scores ≥44)	107	(10.6)		
Overall job stress			22.86	9.14
Scores ≥25	582	(57.7)		
Scores <25	426	(42.3)		
Job satisfaction			67.08	17.22
Scores ≥61	672	(66.7)		
<61	336	(33.3)		
Fear of COVID (emotional reactions)			8.52	4.00
Fear of COVID (symptomatic reactions)			4.34	2.30

### Prevalence of ‘making every contact count’

Results are summarised in Table 1. Healthcare professionals reported that 49.11% of patients whom they saw in a typical week would benefit from health behaviour change interventions. Despite this, healthcare professionals felt able to deliver behaviour change interventions in just 38.00% of such consultations. When behaviour change was discussed with patients, it took on average 26.54% of the consultation time.

### Overall job stress and job satisfaction

The highest levels of perceived job stress were reported by midwives (*M* = 26.10, SD = 10.31) and general practitioners (*M* = 24.91, SD = 8.88). The lowest levels of perceived job stress were reported by specialist doctors (*M* = 21.63, SD = 9.13), ambulance staff (*M* = 21.70, SD = 9.76), and healthcare professionals providing support to clinical staff (*M* = 21.96, SD = 7.71) (reported in Table 2). The highest levels of perceived job satisfaction were reported by nurses working in GP practices (*M* = 77.02, SD = 16.63). The lowest levels of perceived job satisfaction were reported by midwives (*M* = 64.43, SD = 18.27) (reported in Table 2).

**Table 2.** Sample characteristics with respect to perceptions of work-related stress, job satisfaction, impact of events, and fear of COVID-19, by healthcare professional group.

	Impact of events		Overall job stress		Job satisfaction		Fear of COVID (emotional reactions)		Fear of COVID (symptomatic reactions)	
Healthcare professional group	*M*	(SD)	*M*	(SD)	*M*	(SD)	*M*	(SD)	*M*	(SD)
General practitioners	19.37	16.37	24.91	8.88	70.49	19.85	8.47	4.11	4.86	3.22
Specialist doctors	14.27	13.58	21.63	9.13	66.26	15.87	7.54	3.41	3.71	1.64
Nurses and health visitors	22.28	17.84	23.13	9.47	65.45	17.10	8.59	4.10	4.32	2.26
Midwives	19.00	16.56	26.10	10.31	64.43	18.27	7.83	4.38	4.50	2.61
Ambulance staff	16.20	11.77	21.70	9.76	70.50	17.15	7.60	4.25	3.70	1.06
Scientific, therapeutic and technical staff	20.14	16.74	22.48	7.78	67.21	17.00	8.95	4.55	4.56	2.28
Nurses working in GP practices	20.54	17.66	23.41	9.26	77.02	16.63	9.22	4.67	4.70	2.59
Support to clinical staff	23.70	19.67	21.96	7.71	68.50	14.81	9.54	4.55	5.04	2.93
Other HCHS staff/unknown classifications	20.75	15.78	23.64	8.42	65.47	15.69	8.53	3.65	4.52	2.41

### Impact of events

The highest levels of perceived impact of events were reported by healthcare professionals providing support to clinical staff (*M* = 23.70, SD = 19.67), and nurses and health visitors (*M* = 22.28, SD = 17.84). The lowest levels of perceived impact of events were reported by specialist doctors (*M* = 14.27, SD = 13.58) (reported in Table 2). Of the total sample, 31.6% reported scores in subclinical category, 33.5% reported scores in the mild category, 24.2% reported scores in the moderate category, and 10.6% of the sample reported scores in the severe category.

### Fear of COVID-19 (emotional and symptomatic reactions)

The highest levels of perceived fear of COVID-19 (emotional reactions) were reported by healthcare professionals providing support to clinical staff (*M* = 9.54, SD = 4.55) and nurses working in GP practices (*M* = 9.22, SD = 4.67). The lowest levels of perceived fear of COVID-19 (emotional reactions) were reported by specialist doctors (*M* = 7.54, SD = 3.41) and ambulance staff (*M* = 7.60, SD = 4.25) (reported in Table 2). The highest levels of perceived fear of COVID-19 (symptomatic reactions) were reported by healthcare professionals providing support to clinical staff (*M* = 5.04, SD = 2.93). The lowest levels of perceived fear of COVID-19 (symptomatic reactions) were reported by ambulance staff (*M* = 3.70, SD = 1.06) and specialist doctors (*M* = 3.71, SD = 1.64) (reported in Table 2).

### Pearson’s correlations between making every contact count variables, wellbeing and COVID-19-related variables

Table 3 presents the Pearson’s correlations in relation to the making every contact count variables (comprised of: perceptions of the proportion of patients that would benefit from behaviour change interventions, the proportion to whom healthcare professionals deliver interventions, and the amount of time spent on this activity) and COVID-related variables. There were statistically significant correlations across a number of the variables. There are three findings of note. First, the significant positive correlations between perceived impact of events and both emotional and symptomatic reactions to COVID-19 were of a large magnitude (rs > 0.50; Cohen, 1992) as might be expected. Second, perceptions of the number of patients that would benefit from behaviour change interventions were significantly positively correlated with the proportion of times they delivered interventions and how much of their contact time was spent delivering interventions (rs > 0.50). Third, proportion of times interventions were delivered was significantly positively correlated with time spent delivering interventions (*r* = 0.655).

**Table 3.** Pearson’s correlations in relation to making every contact count variables and COVID-related variables.

Variables	Proportion	Delivery	Time	Impact of events	Fear (emotional)	Fear (symptomatic)	Gender	Age	Ethnicity	HCP group	Job stress	Job satisfaction	M	SD
Proportion	1.000	0.707**	0.556**	0.068*	−0.003	−0.014	0.008	0.106**	−0.041	−0.094**	−0.013	0.067*	49.14	35.63
Delivery		1.000	0.655**	0.091**	0.048	0.052	0.047	0.106**	−0.025	−0.094**	0.005	0.099**	37.93	36.34
Time			1.000	0.134**	0.074*	0.089**	0.041	0.090**	−0.004	−0.127**	0.056	0.047	26.54	32.69
Impact of Events				1.000	0.552**	0.547**	0.064*	−0.082**	−0.005	−0.107**	0.419**	−0.246**	20.04	16.83
Fear of COVID (emotional response)					1.000	0.706**	0.054	−0.038	0.063*	−0.013	0.271**	−0.130**	8.52	4.00
Fear of COVID (symptomatic response)						1.000	−0.008	−0.106**	0.064*	0.009	0.290**	−0.133**	4.35	2.30
Gender							1.000	−0.126**	−0.079*	−0.031	0.078*	−0.021	-	-
Age								1.000	−0.140**	−0.178**	−0.130**	0.024	45.23	12.09
Ethnicity									1.000	0.117**	0.058	−0.049	-	-
HCP group										1.000	−0.024	0.076*	-	-
Job stress											1.000	−0.557**	22.86	9.14
Job satisfaction												1.000	67.08	17.22

Proportion: proportion of patients who would benefit; Delivery: delivery of interventions; Time: time spent delivering interventions.

* *p* < 0.05. **p < 0.01. **p < 0.001.

### Associations between sociodemographic and psychosocial variables and delivering behaviour change interventions

Hierarchical linear regression (Table 4, Model 1) showed that impact of events was a positive significant predictor of each of the three outcomes (perceptions of proportion benefitting, proportion receiving and time spent). These effects remained significant when also controlling for demographic variables, health professional group, job stress and job satisfaction (Table 4, Model 2). This suggested that healthcare professionals who were experiencing greater levels of trauma symptoms in response to the events of the COVID-19 pandemic more often reported perceiving patients as benefitting from behaviour change interventions, delivered these in a higher proportion of consultations and spent more time overall delivering behaviour change interventions. Fear of COVID-19 (emotional or symptomatic reactions) was not related to any of the three outcomes in either model.

**Table 4.** Associations between sociodemographic variables, social and psychological variables, and delivering behaviour change interventions.

	Model 1			Model 2		
Variable	*B*	SE	95% CI	*B*	SE	95% CI
Of the service users you see in a typical working week, what proportion do you think would benefit from you making every contact count?						
Impact of events	0.12**	0.08	0.08, 0.41	0.13**	0.09	0.09, 0.44
Fear of COVID (emotional reactions)	−0.03	0.41	−1.06, 0.56	−0.04	0.41	−1.14, 0.48
Fear of COVID (symptomatic reactions)	−0.05	0.72	−2.24, 0.57	−0.04	0.72	−1.96, 0.86
Gender (1 = Men; 2 = Women)				0.01	2.59	−4.18, 5.99
Age				0.10**	0.10	0.11, 0.49
Ethnicity (1 = White; 2 = Black, Asian or Minority Ethnic)				−0.01	3.57	−7.64, 6.39
Healthcare professional group (1 = nurse, health visitor; 2 = other)				−0.07*	2.36	−9.60, −0.36
Overall job stress				0.03	0.16	−0.21, 0.41
Job satisfaction				0.10**	0.08	0.06, 0.37
Of the service users you see in a typical working week, who you think would benefit, with what proportion do you make every contact count?						
Impact of events	0.09*	0.08	0.04, 0.37	0.10*	0.09	0.05, 0.40
Fear of COVID (emotional reactions)	−0.02	0.42	−0.97, 0.68	−0.03	0.42	−1.12, 0.52
Fear of COVID (symptomatic reactions)	0.02	0.73	−1.18, 1.68	0.04	0.73	−0.82, 2.03
Gender (1 = Men; 2 = Women)				0.05	2.62	−0.62, 9.66
Age				0.12***	0.10	0.16, 0.54
Ethnicity (1 = White; 2 = Black, Asian or Minority Ethnic)				0.01	3.61	−5.81, 8.37
Healthcare professional group (1 = nurse, health visitor; 2 = other)				−0.07*	2.38	−10.08, −0.74
Overall job stress				0.06	0.16	−0.07, 0.56
Job satisfaction				0.17***	0.08	0.19, 0.50
Of the service users you see in a typical working week who you think would benefit, how much of their appointment time do you spend with them making every contact count?						
Impact of events	0.13**	0.08	0.10, 0.40	0.12**	0.08	0.07, 0.38
Fear of COVID (emotional reactions)	−0.02	0.38	−0.89, 0.60	−0.03	0.37	-1.00, 0.46
Fear of COVID (symptomatic reactions)	0.04	0.65	−0.78, 1.79	0.06	0.65	−0.49, 2.06
Gender (1 = Men; 2 = Women)				0.04	2.35	−1.29, 7.94
Age				0.10**	0.09	0.11, 0.45
Ethnicity (1 = White; 2 = Black, Asian or minority ethnic)				0.03	3.24	−3.39, 9.34
Healthcare professional group (1 = nurse, health visitor; 2 = other)				−0.11**	2.14	−11.49, −3.10
Overall job stress				0.08*	0.14	0.02, 0.58
Job satisfaction				0.13***	0.07	0.12, 0.39

* *p*  <  0.05. **p  <  0.01. ***p  <  0.001.

Table 4, Model 2 also showed age (positively), healthcare professional group (negatively) and job satisfaction (positively) to be significant predictors of the three outcomes. This suggested that older, more satisfied healthcare professionals and nurses/health visitors more often reported perceiving patients as benefitting from behaviour change interventions, delivered these in a higher proportion of consultations and spent more time overall delivering behaviour change interventions. Job stress was a significant positive predictor of time spent on making every contact count, but not the other two outcomes (Table 4, Model 2). Gender and ethnicity were unrelated to any of the three outcomes.

Entering interactions in Model 3 (not shown in Table 4) indicated only one significant interaction (impact of events x age on perceptions of patient benefit; [*B* = 0.011, SE = 0.006, *p* = 0.048]). Simple slope analyses indicated that as age increases from low (*M* −1 SD) to moderate (*M*) to high (*M* + 1 SD) the impact of events increases from *B* = 0.014, SE = 0.021, *p* = 0.495 to *B* = 0.036, SE = 0.015, *p* = 0.015 to *B* = 0.059, SE = 0.021, *p* = 0.005. This indicates that impact of events had a non-significant effect in younger participants but had a significant positive effect in participants of a mean age and a strong significant positive effect in older participants.

## Discussion

### Principal findings

The present research assessed the extent to which healthcare professional characteristics and perceptions of a public health emergency were associated with delivering health behaviour change interventions during routine consultations. Older respondents, higher levels of job satisfaction, being a nurse or health visitors, and reporting higher levels of perceived impacts of the COVID-19 public health emergency were associated with higher proportions of perceived patient benefit, higher prevalence of delivering behaviour change interventions, and greater amount of reported time delivering interventions. Higher levels of emotional job stress were associated with greater time spent delivering interventions (but not intervention delivery). Interventions targeted at younger healthcare professionals, those reporting lower job satisfaction, and healthcare professionals other than nurses or health visitors may be particularly beneficial.

### Comparison with previous literature

Consistent with previous literature (Labrague and de Los Santos, 2021) our findings suggest that perceived fear of COVID-19 in healthcare professionals is negatively related to job satisfaction, which may suggest an important role for organisations to support the mental health and wellbeing of healthcare staff in the recovery from public health emergencies (Keyworth et al., 2022; Shanafelt et al., 2020). Further, according to healthcare professional group, the highest levels of perceived job stress were reported amongst midwives, which is consistent with previous research conducted amongst midwives showing high levels of negative psychological effects because of the effects of public health emergencies, including prevalence of post-traumatic stress disorder, stress, and anxiety (Couper et al., 2022). We also found comparable levels of perceived job stress amongst GPs, suggesting this may be particularly prominent amongst patient facing healthcare staff, which has been demonstrated in the literature (Arora et al., 2022).

Research suggests that the negative impacts of COVID-19 specifically may result in reduced job satisfaction amongst healthcare professionals (Çağış and Yıldırım, 2023) which may translate into reduced work performance more broadly, consequently leading to higher levels of job dissatisfaction and increased intentions to leave the profession and the organisation (Pathman et al., 2002; Windover et al., 2018). Our findings suggest this may also translate into specific areas of professional practice; our findings show that increased job satisfaction is associated with intervention delivery and time spent delivering interventions. This association may be indicative of a broader relationship where job satisfaction not only impacts the likelihood of delivering interventions but may also influence the overall quality and effectiveness of these interventions. For instance, higher job satisfaction might enhance a healthcare professional’s engagement and motivation to deliver health behaviour change interventions, leading to more thorough and impactful delivery of interventions. It would be valuable to examine whether this translates into other areas of healthcare professional practice, particularly in areas that would be beneficial for patient care such as routine health screening (Stevens et al., 2019). However, contrary to predictions, our findings suggest that higher perceived impacts of COVID-19 translated into higher proportions of perceived patient benefit, higher prevalence of delivering behaviour change interventions, and greater amount of reported time delivering interventions. Whilst previous research suggests the negative impacts of public health emergencies may have negative psychological consequences for healthcare professionals (Kisely et al., 2020; Kobres et al., 2019; Mitchell et al., 2022; Wilder-Smith and Osman, 2020), our findings suggest perceptions may have positive effects on delivering behaviour change interventions. One possible explanation from the broader literature may be that some level of workplace anxiety may facilitate job performance (Cheng and McCarthy, 2018).

### Implications

Our findings suggest several important targets for interventions aimed at supporting healthcare professionals during and following public health emergencies. It would be valuable to explore whether increasing job satisfaction amongst healthcare professionals results in greater delivery of behaviour change interventions. Higher levels of job satisfaction have been shown to have positive effects on some areas of work performance such as higher quality of patient care, greater patient adherence to treatments, and higher levels of patient satisfaction (Scheepers et al., 2015; Williams and Skinner, 2003). Our findings suggest this may have the potential to translate to other areas of practice. More broadly, research shows overall job satisfaction of healthcare workers has declined over the last decade (Ocean and Meyer, 2023) which is a cause for concern, particularly given that job dissatisfaction may predict intention to leave the healthcare sector (Pathman et al., 2002).

### Strengths and limitations

To our knowledge, this is the first study to examine the extent to which perceptions of a public health emergency is associated with healthcare professional delivery of health behaviour change interventions within a representative sample of NHS healthcare professionals, highlighting key intervention targets. Findings also highlight opportunities to support healthcare professionals to identify patient need for interventions and to support intervention delivery as part of routine consultations. However there are several limitations to this study. The sample may not be fully representative of the healthcare professionals working in the NHS as a whole. However, YouGov attempted to overcome this by seeking the widest possible variation in participants according to demographic characteristics. Additionally the cross-sectional nature of the study meant that we were unable to track any changes over time in our respondents, and causality cannot be inferred.

### Future research

Further work is needed to increase the delivery of behaviour change interventions by healthcare professionals in the recovery from public health emergencies. In particular, our research provides insights into groups that would benefit most from such interventions: younger healthcare professionals, those reporting lower job satisfaction, and healthcare professionals other than nurses or health visitors. Specific interventions should capitalise on the opportunity to deliver brief interventions to a population who have frequent contact with patients and therefore in a unique position to influence population health on a wide scale (Keyworth et al., 2018, 2019). For example, our previous research showed healthcare professionals reported lower levels of automatic motivation (i.e. delivering intervention through habit), compared to the other five domains of the capability, opportunity, and motivation model of behaviour (Keyworth et al., 2024; Michie et al., 2011). One possible approach could be to encourage healthcare professionals to use implementation intentions (Gollwitzer, 1993), which have been shown to be effective in changing behaviour (Armitage, 2016; Armitage et al., 2014), by linking critical situations with an appropriate response. However, despite being brief enough to be deployed at scale with high public health ‘reach’ in healthcare settings, these have rarely been used in the context of healthcare professional behaviour change.

## Conclusions

Delivering behaviour change interventions is an important area of healthcare professional routine practice. Our research suggests there are opportunities to increase delivery of interventions with more targeted efforts. Interventions targeted at younger healthcare professionals, those reporting lower job satisfaction, and healthcare professionals other than nurses or health visitors should be prioritised.
